# VETERANS WITH CAREGIVERS UNDER AGE 25: IMPORTANT DISTINCTIONS FOR RESEARCH, POLICY, AND PRACTICE

**DOI:** 10.1093/geroni/igae098.1094

**Published:** 2024-12-31

**Authors:** Andrea Kalvesmaki, Sandra Garcia-Davis, Erin Bouldin, Ranak Trivedi, Luci Leykum, Stuti Dang

**Affiliations:** VA Salt Lake City Health Care System, Salt Lake City, Utah, United States; University of Miami, Miami, Florida, United States; University of Utah, Salt Lake City, Utah, United States; Stanford University/VA Palo Alto Healthcare System, Palo Alto, California, United States; South Texas Veterans Health Care System, Austin, Texas, United States; Miami VA Healthcare System, Miami, Florida, United States

## Abstract

“Young caregivers” are those aged 25 or less. Up to 5 million young caregivers under the age of 18 provide secondary care for an aging adult, which doubles if including those up to age 25. However, these caregivers are underrecognized in research and as such, may have many unmet needs, along with their care recipients. This project represents a secondary analysis of the HERO CARE survey in which we collected information on up to three additional caregivers supporting the Veterans in our study. Our goal was to identify Veterans with caregivers under the age of 25 and compared differences in socio-demographic characteristics and unmet needs to Veterans with caregivers over the age of 25, and Veterans without caregivers using chi-square and analysis of variance (ANOVA) tests. One hundred and fifty-four respondents indicated they had at least one caregiver younger than 25. Preliminary findings showed that, compared to both Veterans without a caregiver (n=2574) and those with caregivers older than 25 years (n=4027), Veterans with caregivers younger than age 25 tended to have lower education, have medication insecurity, food insecurity, low health literacy, transportation problems, have more unmet needs with daily activities, and be Black or Hispanic (p’s<0.05). Findings suggest that the presence of a young caregiver may reflect greater social vulnerability for Veterans, and future research should focus on the risk of young caregivers for poor mental health, physical health, educational and economic outcomes.

